# 95347 Examining the Impact of the BPCA: Promoting Pediatric Inclusion in Clinical Trials and Pediatric-Specific Drug Information

**DOI:** 10.1017/cts.2021.673

**Published:** 2021-03-30

**Authors:** Annie Ly, Terry Church

**Affiliations:** 1 Candidate of Regulatory Science, University of Southern California; 2 University of Soutern California

## Abstract

ABSTRACT IMPACT: It provides insight in the relationship between pediatrics and clinical research and how pediatric participation in CT translates to clinical significance in form of drug labels, which inform clinicians on how to prescribe pediatric medications. OBJECTIVES/GOALS: Assessing the extent that the Best Pharmaceuticals for Children Act (BPCA) advances pediatric inclusion in clinical trials (CTs) and the availability of pediatric-specific drug information METHODS/STUDY POPULATION: The BPCA provides the U.S. Food and Drug Administration (FDA) authority to solicit sponsors whose drugs may benefit pediatric populations. Participation is voluntary and provides additional market exclusivity and pediatric information. CTs that received marketing exclusivity from 2016-2018 under BPCA were reviewed using Clinicaltrials.gov to access the legislation’s impact. CTs were categorized according to eligibility: (1) pediatric and adult groups, (2) pediatrics, and (3) pediatric sub-groups. Studies were excluded for ambiguous age data. Studies open to both groups were evaluated for pediatric participation. Each drug was searched in DailyMed.com for published pediatric indications. RESULTS/ANTICIPATED RESULTS: Between 2016 - 2018, 22 drugs received marketing exclusivity under BPCA. Of the 196 CTs conducted for these drugs, 135 were available to adults and pediatrics, 10 were available to the entire pediatric population, and 51 were available to specific pediatric sub-populations. Exclusion criteria permitted only 118 of the CTs for assessment where eligibility included both pediatric and adult populations, of which 65 of these had less than 1% pediatric representation. Of the 22 drugs, 20 have pediatric indications. Over this three-year period, the number of CTs where adults and pediatrics were eligible were greater than CTs for pediatric only or pediatric subpopulations. DISCUSSION/SIGNIFICANCE OF FINDINGS: It is prevalent for BPCA compliant CTs to include both; 65% of drugs (13/20) with pediatric indications had more studies involving both groups than only pediatrics. Adequate pediatric CT representation is necessary for developing pediatric drug labeling with meaningful data for clinical indications.

